# Association of Electronic Health Record Design and Use Factors With Clinician Stress and Burnout

**DOI:** 10.1001/jamanetworkopen.2019.9609

**Published:** 2019-08-16

**Authors:** Philip J. Kroth, Nancy Morioka-Douglas, Sharry Veres, Stewart Babbott, Sara Poplau, Fares Qeadan, Carolyn Parshall, Kathryne Corrigan, Mark Linzer

**Affiliations:** 1 University of New Mexico, Albuquerque; 2 Stanford University, Palo Alto, California; 3 Centura Health, Westminster, Colorado; 4 University of Virginia, Charlottesville; 5 Minneapolis Medical Research Foundation, Minneapolis, Minnesota; 6 University of Utah, Salt Lake City; 7 Uniformed Services University of the Health Sciences, Bethesda, Maryland; 8 Hennepin County Medical Center, Minneapolis, Minnesota

## Abstract

**Importance:**

Many believe a major cause of the epidemic of clinician burnout is poorly designed electronic health records (EHRs).

**Objectives:**

To determine which EHR design and use factors are associated with clinician stress and burnout and to identify other sources that contribute to this problem.

**Design, Setting, and Participants:**

This survey study of 282 ambulatory primary care and subspecialty clinicians from 3 institutions measured stress and burnout, opinions on EHR design and use factors, and helpful coping strategies. Linear and logistic regressions were used to estimate associations of work conditions with stress on a continuous scale and burnout as a binary outcome from an ordered categorical scale. The survey was conducted between August 2016 and July 2017, with data analyzed from January 2019 to May 2019.

**Main Outcomes and Measures:**

Clinician stress and burnout as measured with validated questions, the EHR design and use factors identified by clinicians as most associated with stress and burnout, and measures of clinician working conditions.

**Results:**

Of 640 clinicians, 282 (44.1%) responded. Of these, 241 (85.5%) were physicians, 160 (56.7%) were women, and 193 (68.4%) worked in primary care. The most prevalent concerns about EHR design and use were excessive data entry requirements (245 [86.9%]), long cut-and-pasted notes (212 [75.2%]), inaccessibility of information from multiple institutions (206 [73.1%]), notes geared toward billing (206 [73.1%]), interference with work-life balance (178 [63.1%]), and problems with posture (144 [51.1%]) and pain (134 [47.5%]) attributed to the use of EHRs. Overall, EHR design and use factors accounted for 12.5% of variance in measures of stress and 6.8% of variance in measures of burnout. Work conditions, including EHR use and design factors, accounted for 58.1% of variance in stress; key work conditions were office atmospheres (*β̂* = 1.26; *P* < .001), control of workload (for optimal control: *β̂* = −7.86; *P* < .001), and physical symptoms attributed to EHR use (*β̂* = 1.29; *P* < .001). Work conditions accounted for 36.2% of variance in burnout, where challenges included chaos (adjusted odds ratio, 1.39; 95% CI, 1.10-1.75; *P* = .006) and physical symptoms perceived to be from EHR use (adjusted odds ratio, 2.01; 95% CI, 1.48-2.74; *P* < .001). Coping strategies were associated with only 2.4% of the variability in stress and 1.7% of the variability in burnout.

**Conclusions and Relevance:**

Although EHR design and use factors are associated with clinician stress and burnout, other challenges, such as chaotic clinic atmospheres and workload control, explain considerably more of the variance in these adverse clinician outcomes.

## Key Points

**Question:**

Which electronic health record (EHR) design and use factors are associated with clinician stress and burnout?

**Findings:**

In this survey study of 282 clinicians, clinician stress and burnout were associated with 7 EHR design and use factors. These 7 plus 2 other design and use factors collectively accounted for a modest amount of the variance in stress (12.5%) and burnout (6.8%); models incorporating other work conditions (such as chaotic work atmosphere and workload control) accounted for considerably more of the variance in stress (58.1%) and burnout (36.2%).

**Meaning:**

While EHR design and use factors may appropriately be targeted by health systems and EHR designers to address stress and burnout, other non-EHR issues, especially clinician work conditions, appear to play a substantial role in adverse clinician outcomes.

## Teaser

This survey study determines which electronic health record (EHR) design and use factors are associated with clinician stress and burnout and identifies other sources that contribute to this problem.

## Introduction

The adoption of the electronic health record (EHR) has occurred alongside the dramatic and troubling rise in clinician stress and burnout.^1,2,3^ This association has fueled the debate over the extent to which EHRs are associated with the epidemic of clinician stress and burnout. Technostress (ie, the stress related to technological tools in numerous industries) is real,^4^ but the degree to which it is a factor in medicine is largely unknown.

The introduction of EHRs has resulted in shifting many clerical tasks to clinicians (eg, billing, coding, and quality control) as well as creating new tasks to be performed during clinical encounters (eg, data entry, computerized decision support, computerized order entry, and electronic prescribing). These new tasks have increased the cognitive and physical load on the clinician in many ways.^5,6^ For example, e-prescribing, which has benefits, has also created an additional burden by requiring clinicians to know where to route prescriptions at the time they prescribe. This may be a relatively small burden, but repeated multiple times per day and added to the myriad other tasks shifted to clinicians, these technology-enabled tasks have considerably increased clinician workload. In fact, an entirely new medical scribe industry has arisen in order to ameliorate the additional workload.^7^

We designed this study (Minimizing Stress, Maximizing Success of the Electronic Health Record) to identify the relative contribution of aggregated EHR burdens compared with other burdens (ie, workplace chaos, control of workload) associated with clinician stress and burnout. This work is based on a conceptual framework derived from prior work (Figure).^8^ Our hypothesis was that EHR-associated stress adds to overall stress and could lead to burnout—which may play a role in the quality of patient care. In this study, we aim to understand which EHR design and use factors are associated with stress and burnout. The potentially challenging EHR design and use factors included in the survey instrument were identified through physician focus groups conducted in the first phase of the study.^9^ The design and use factors studied were intentionally limited to those over which clinicians and their institutions might have some control. This in no way minimizes other societal factors, such as governmental regulation and malpractice, that could be associated with clinician stress and burnout.^10,11,12^ This survey phase of our study quantifies the association of these EHR design and use factors with clinician stress and burnout to address the following questions: (1) what specific EHR design and use factors are most strongly associated with clinician stress and burnout? (2) What amount of overall stress and burnout is associated with EHRs? And, (3) what coping strategies or organizational solutions did respondents feel are important in addressing stress and burnout?

**Figure.**
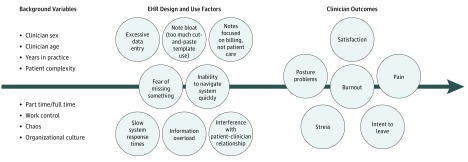
Conceptual Framework of Association of Work Conditions and Electronic Health Record (EHR) Design and Use Factors With Clinician Outcomes

## Methods

### Identification of Challenging EHR Design and Use Factors

The methods for this study have been previously reported.^9^ In brief, physician focus groups at 3 institutions (Stanford Hospital and Clinics, Stanford, California; University of New Mexico, Albuquerque; and Centura Health Physician Group, Westminster, Colorado) identified EHR design and use factors that were perceived as successful and those that were associated with user stress, burnout, or unintended physical symptoms. We also identified commonly used coping strategies by the clinicians.

### Survey and Sampling

The EHR design and use factors identified in prior clinician focus groups informed the design of the survey instrument, which is freely available.^13^ The instrument included questions from previously validated instruments to measure stress, burnout, and other challenges identified by Motowidlo,^14^ the Physician Worklife Survey,^15^ the Minimizing Error, Maximizing Outcome Study,^16^ and the Healthy Work Place Study.^17,18^ Questions also focused on workplace characteristics such as workload control^19^ and work atmosphere (a single item measure from the Minimizing Error, Maximizing Outcome Study)^20^ as well as patient complexity and organizational culture, including value alignment between leaders and clinicians. This survey study complied with the American Association for Public Opinion Research (AAPOR) reporting guideline.

The study survey instrument was pilot tested on 10 clinicians at Hennepin County Medical Center (Minneapolis, Minnesota). We then deployed the finalized instrument in 2 waves at the 3 focus group sites from August 9, 2016, through July 7, 2017. The institutional review boards at all participating institutions approved the study, and completing the survey was considered providing consent.

We used REDCap version 8.10.7 (Vanderbilt University) to deploy an electronic version of the instrument. Nonresponders to the REDCap electronic survey were mailed paper instruments. The electronic instrument used continuous slider bars for respondents to indicate a score from 0 to 100, where 0 indicated not at all and 100 indicated to a great extent. The paper instrument used Likert scales mapped to the scale of 0 to 100 for analysis (ie, 1, not at all, mapped to 15; 2 mapped to 40; 3 mapped to 60; and 4, to a great extent, mapped to 85).

The survey’s design attempted to determine the following: (1) perceived EHR successes, (2) EHR design and use factors associated with clinician stress and burnout, (3) perceived adverse personal outcomes (eg pain or anxiety), (4) things that could improve the EHR experience (eg, greater staff support, scribes, or fewer clicks per task), and (5) coping strategies (eg, exercise or setting boundaries). We sampled clinicians (physicians and advanced practice clinicians, including nurse practitioners and physician assistants) at 3 institutions from 5 disciplines: general internal medicine, medical subspecialties, general pediatrics, pediatric subspecialties, and family medicine. We excluded residents, as we thought they could have dissimilar experiences of stress and burnout than practicing clinicians. We determined respondent stress levels using the 4-item validated measures from Motowidlo,^14^ a continuous measure that ranges from 4 to 20, and burnout using the single-item validated measure from the Physician Worklife Study, in which a score of 3 or more indicates burnout.^21^ While a binary approach to burnout has been controversial,^22,23^ this measure has been used and validated in many settings and among thousands of respondents for 20 years, and it is associated with adverse work conditions and adverse clinician outcomes, such as intent to leave the practice. We ran additional analyses using the 5-choice measure of burnout as an ordered categorical (as opposed to binary) outcome and found no substantive differences between the 2 methods.

### Statistical Analysis

Answers to survey questions were analyzed as standard summary statistics. We reported continuous variables as mean and SD and categorical variables as number of respondents and percentages of total sample.

Linear regression was used to determine the association of focus group–identified variables (eg, work conditions, EHR design and use factors, and coping strategies) with clinician-reported stress, which we scored according to the Motowidlo 4-item measure,^14^ and burnout. *β̂* was used to estimate the magnitude and direction of association, and it was calculated using the least-square estimation technique. We used logistic regression with stepwise selection, which is a combination of the forward and backward selection techniques, to estimate the association of focus group–identified variables with the odds of clinician-reported burnout, which we measured as a binary outcome based on a single question (with burnout representing endorsement of any choice with the word burnout in it).^14^ We used construct variables created to summarize the associations of variables within the same domain with stress and burnout. To develop the final regression model for stress, variables with *R*^2^ greater than 0.10 in the univariate analysis or that were determined to be of special interest were considered candidate variables for the multivariable model. The final logistic regression model for burnout used a stepwise selection technique, which was determined to be the most comprehensive method because it combines both forward and backward selection. To justify lumping together different types of clinicians and specialties, 1-way analysis of variance was used to examine if statistically significant differences existed in the means of outcome measures across clinician type (ie, MD, DO, nurse practitioner, or physician assistant) or specialty (ie, primary care, nonprocedural specialist, or procedural specialist). Diagnostics done on the regression and logistic models were the Breusch-Pagan test for constant variance and the Hosmer-Lemeshow test for goodness of fit, noting that *P* > .05 indicates having constant variance for the regression model and correct fit for the logistic model respectively. (These showed that the models were well calibrated.) Finally, we performed a statistical factor analysis using the varimax rotation method on 9 EHR design and use items to summarize the association of EHRs with stress and burnout. We used SAS version 9.4 (SAS Institute, Inc) for all analyses. Statistical significance was set at *P* < .05, and all tests were 2-tailed.

## Results

### Sample and Work-Life Balance Description

Between August 2016 and July 2017, we surveyed 640 clinicians from 3 institutions, with 282 (44.1%) responding (208 [73.8%] electronically and 74 [26.2%] on paper); 160 (56.7%) were women, 241 (85.5%) were physicians (MDs and DOs), and 193 (68.4%) worked in primary care (Table 1). Overall, 256 respondents (90.8%) answered at least 95 of the 105 survey questions. The 1-way analysis of variance showed no significant difference in mean (SD) burnout between clinician types (DO, 2.33 [0.52]; MD, 2.54 [0.94]; nurse practitioner, 2.14 [0.53]; physician assistant, 2.45 [0.94]; *P* = .42) or between practice types (primary care, 2.51 [0.52]; nonprocedural specialist, 2.48 [0.82]; procedural specialist 2.59 [0.76]; *P* = .86). Therefore, neither of these components was controlled for in the analysis. Most participants noted stressful work conditions: 210 (74.5%) reported time pressure for documentation, and 170 (60.2%) spent moderately high or excessive time on the EHR at home (Table 1). Overall, 142 (50.4%) felt they had insufficient personal time, and 134 (47.5%) reported having minimal coverage for their EHR inboxes when needed. Only 95 (33.7%) reported that their practices emphasized work-life balance, while 215 (76.2%) said that productivity was overemphasized. Half (140 [49.6%]) reported marginal or poor control over workload, and 143 (50.7%) judged their office atmospheres as chaotic or tending toward chaotic. Almost half (127 [45.0%]) described symptoms of burnout, and 117 (41.5%) indicated they were moderately to definitely likely to leave their practices within 2 years (Table 1).

**Table 1.** Respondent Demographic Characteristics

Characteristic	No. (%)
Age, mean (SD), y	50 (11)
NR	5 (1.8)
Sex	
Male	118 (41.8)
Female	160 (56.7)
NR	46 (16.3)
Race/ethnicity	
Hispanic, any race	30 (10.6)
White non-Hispanic	191 (67.7)
Asian	43 (15.3)
Other or NR^a^	18 (6.4)
Clinician type	
MD	241 (85.5)
PA	20 (7.1)
NP	14 (5.0)
DO	6 (2.1)
NR	1 (0.4)
Practice type	
Primary care	193 (68.4)
Nonprocedural specialist	53 (18.8)
Procedural specialist	28 (9.9)
Multiple practice types	5 (1.8)
Not specified	45 (13.0)
NR	3 (1.1)
Roles	
Full time	226 (80.1)
Part time	54 (19.1)
NR	2 (0.7)
% of patients, mean (SD) [NR]	
With ≥3 complex medical problems	64.4 (27.2) [0]
With complex psychosocial problems	50.4 (26.8) [1]
Non-English speaking	18.8 (18.4) [0]
Likelihood of leaving practice in 2 y	
None	75 (26.6)
Slight	89 (31.6)
Moderate	59 (20.9)
Likely	36 (12.8)
Definitely	22 (7.8)
NR	1 (0.4)
Enough time for personal and family life	
Strongly disagree	50 (17.7)
Disagree	92 (32.6)
Neither agree nor disagree	54 (19.2)
Agree	78 (27.7)
Strongly agree	8 (2.8)
NR	0
Inbox coverage when out of office	
Slight or none	62 (22.0)
Some	72 (25.5)
Moderate	78 (27.7)
Great	68 (24.1)
NR	2 (0.7)
Enough time for charting at work	
Poor	86 (30.5)
Marginal	124 (44.0)
Satisfactory	51 (18.1)
Good	17 (6.0)
Optimal	1 (0.4)
NR	3 (1.1)
Time spent on EHR at home	
Excessive	61 (21.6)
Moderately high	109 (38.7)
Satisfactory	20 (7.1)
Modest	40 (14.2)
Minimal or none	52 (18.4)
NR	0
Workplace emphasizes work-life balance	
Slight or none	74 (26.2)
Some	113 (40.1)
Moderate	77 (27.3)
Great	18 (6.4)
NR	0
Workplace emphasizes productivity	
Slight or none	11 (3.9)
Some	56 (19.9)
Moderate	131 (46.5)
Great	84 (29.8)
NR	0
Control over workload	
Poor	46 (16.3)
Marginal	94 (33.3)
Satisfactory	94 (33.3)
Good	44 (15.6)
Optimal	4 (1.4)
NR	0
Office atmosphere	
Calm	5 (1.8)
Tending to be busy	15 (5.3)
Busy, but reasonable	77 (27.3)
Tending to be chaotic	110 (39.0)
Hectic, chaotic	33 (11.7)
NR	42 (14.9)
Symptoms of burnout	
No symptoms	28 (9.9)
Occasionally stressed but not burned out	126 (44.7)
Burning out with ≥1 symptom	94 (33.3)
Burnout symptoms will not go away	22 (7.8)
Completely burned out and wonder if I can go on	11 (3.9)
NR	1 (0.4)

Abbreviations: EHR, electronic health record; NP, nurse practitioner; NR, no response; PA, physician assistant.

^a^ Other category included Native American or Alaska Native, Native Hawaiian or Pacific Islander, Black or African American, or other.

### Success and Challenges of the EHR

The EHR successes participants identified included the ability to message colleagues electronically (197 [69.9%]), access to the EHR from home (213 [75.5%]), and the opportunity to share results with patients (136 [48.2%]). The most troublesome EHR design and use factors reported were excessive data entry requirements (245 [86.9%]), “note bloat” (unnecessarily long cut-and-pasted progress notes; 212 [75.2%]), inaccessible information from other institutions (206 [73.1%]), notes geared toward billing rather than patient care (206 [73.1%]), problems with work-life balance (178 [63.1%]), and 2 physical items that respondents attributed to EHR use: posture issues (144 [51.1%]) and pain (134 [47.5%]).

### Association of EHR Use and Design Factors With Stress and Burnout

The EHR design and use factors significantly associated with high clinician stress were information overload (*β̂* = 0.37; *P* < .001), slow system response times (*β̂* = 0.42; *P* < .001), excessive data entry (*β̂* = 0.43; *P* < .001), inability to navigate the system quickly (*β̂* = 0.38; *P* < .001), note bloat (*β̂* = 0.24; *P* = .01), fear of missing something (*β̂* = 0.34; *P* < .001), interference with the patient-clinician relationship (*β̂* = 0.29; *P* < .01), and notes geared toward billing (*β̂* = 0.41; *P* < .001) (Table 2). In our analyses, burnout was used as a dichotomous as well as an ordered categorical variable, and there were no substantive differences between the 2 approaches. All of the previously listed EHR design and use factors were independently associated with burnout except fear of missing something. These factors collectively accounted for 12.5% and 6.8% of the variance in stress and burnout (as a binary outcome), respectively. Physical symptoms attributed to EHR use increased odds of burnout (adjusted odds ratio [aOR], 2.01; 95% CI, 1.48-2.75; *P* < .001)

**Table 2.** Design and Use Factors of EHRs Associated With Stress and Burnout

Design and Use Factor^a^	Stress, Continuous			Burnout, Binary			
	*β̂*^b^	*P* Value	*R*^2^, %^c^	OR (95% CI)	*P* Value	AUC	*R*^2^, %^c^
How challenging are the following aspects of your EHR?							
Information overload	0.37	<.001	6.2	1.18 (1.06-1.30)	.002	0.61	5.1
Lack of access to patient information from multiple institutions	0.14	.08	1.1	0.99 (0.91-1.08)	.85	0.50	0.1
Slow system response times	0.42	<.001	8.9	1.13 (1.03-1.24)	.01	0.59	3.2
Excessive data entry	0.43	<.001	6.8	1.24 (1.10-1.40)	<.001	0.65	6.3
Inability to navigate the system quickly	0.38	<.001	6.7	1.12 (1.02-1.24)	.02	0.59	2.7
Note bloat, ie, progress notes too complex to read	0.24	.01	2.4	1.16 (1.04-1.28)	.006	0.60	3.7
Fear of missing something	0.34	<.001	5.4	1.06 (0.96-1.17)	.22	0.55	0.8
Interference with the patient-clinician relationship	0.29	.002	3.7	1.14 (1.03-1.27)	.01	0.60	3.3
Notes geared toward billing not patient care	0.41	<.001	8.8	1.26 (1.14-1.40)	<.001	0.67	10.6
EHR challenges construct variable, per 10-unit increase^d^	0.80	<.001	12.5	1.35 (1.15-1.58)	<.001	0.64	6.8

Abbreviations: AUC, area under the receiving operating characteristic curve; EHR, electronic health record; OR, odds ratio.

^a^ Each factor has the possible value of 0 to 100, where 0 indicates not challenging at all and 100 indicates challenging to a great extent.

^b^ Indicates the rate of change in the EHR challenges construct variable per 10-unit increase in the independent variable.

^c^ Percentage of variability in the primary outcome explained by the design and use factor.

^d^ Created by averaging the response values for all questions yielding a possible range from 0 to 100 for the construct score.

### Other Factors Associated With Stress and Burnout

Factors not related to EHRs associated with high levels of variance in stress were office atmospheres (*β̂* = 1.26; *P* < .001), control of workload (for optimal control: *β̂* = −7.86; *P* < .001), time for personal and family life (for disagree: *β̂* = −2.30; *P* < .001), time for documentation at work (for satisfactory: *β̂* = −2.93; *P* < .001), value alignment with leaders (for agree strongly: *β̂* = −4.73; *P* < .001), professional and personal life balance (*β̂* = −1.56; *P* < .001), physical symptoms attributed to EHR use (*β̂* = 1.29; *P* < .001) and hours worked per week (*β̂* = 0.78; *P* < .001). Within a multivariable linear regression model (Table 3), these variables, along with the EHR design and use factors listed in Table 2, consequences of EHR use, and EHR use at home, accounted for 58.1% of variance in clinician-reported stress and 36.2% of variance in burnout (Table 4). A chaotic work environment increased the odds of burnout (aOR, 1.39; 95% CI, 1.10-1.75; *P* = .006).

**Table 3.** Univariate and Multivariable Models for Stress

Factor	Univariate Models			Multivariable Model	
	Unadjusted* β̂*^a^	*P* Value	*R*^2^, %^b^	Adjusted *β̂*^a^	*P* Value
EHR challenges construct variable, per 10-unit increase	0.80	<.001	12.5	0.13	.36
Office atmosphere, per 10-unit increase^c^	1.26	<.001	34.3	0.66	<.001
Workload control	NA	<.001	28.7	NA	.01
Poor	1 [Reference]	NA	NA	1 [Reference]	NA
Marginal	−1.40	.01	NA	0.22	.71
Satisfactory	−4.25	<.001	NA	−1.06	.14
Good	−5.16	<.001	NA	−1.18	.17
Optimal	−7.86	<.001	NA	−5.48	.004
Work schedule leaves enough time for my personal and family life	NA	<.001	24.0	NA	.10
Strongly disagree	1 [Reference]	NA	NA	1 [Reference]	NA
Disagree	−2.30	<.001	NA	−0.05	.94
Neither agree nor disagree	−4.62	<.001	NA	−1.69	.03
Agree	−4.54	<.001	NA	−5.73	.49
Strongly agree	−6.23	<.001	NA	−6.26	.67
Compensated roles	NA	.14	0.78	NA	.74
Part-time	1 [Reference]	NA	NA	1 [Reference]	NA
Full-time	0.83	.14	NA	−0.21	.74
Sufficiency of time for documentation at work	NA	<.001	12.69	NA	.50
Poor	1 [Reference]	NA	NA	1 [Reference]	NA
Marginal	−1.99	<.001	NA	−0.53	.32
Satisfactory	−2.93	<.001	NA	−0.58	.43
Good	−4.48	<.001	NA	−0.16	.90
Optimal	−2.72	.43	NA	3.70	.21
Professional values are well aligned with those of departmental or clinical leaders	NA	<.001	11.45	NA	.62
Strongly disagree	1 [Reference]	NA	NA	1 [Reference]	NA
Disagree	−2.33	.03	NA	−1.12	.20
Neither agree nor disagree	−2.93	.005	NA	−1.05	.26
Agree	−4.44	<.001	NA	−1.34	.11
Agree strongly	−4.73	<.001	NA	−1.09	.29
Professional and personal life balance, per 1-unit increase	−1.56	<.001	14.06	−0.40	.12
Consequences construct variable, per 100-unit increase^d^	1.29	<.001	22.53	0.69	<.001
Amount of time spent on EHR at home	NA	<.001	8.26	NA	.58
Excessive	1 [Reference]	NA	NA	1 [Reference]	NA
Moderately high	−2.18	<.001	NA	−0.10	.86
Satisfactory	−3.08	<.001	NA	−0.92	.37
Modest	−2.86	<.001	NA	0.50	.51
Minimal or none	−2.51	<.001	NA	0.54	.48
Total average hours worked per week, per 10-unit increase	0.78	<.001	8.25	−0.01	.96

Abbreviations: EHR, electronic health record; NA, not applicable.

^a^ Indicates the rate of change in the EHR challenges construct variable by 1-, 10-, or 100-unit increase in the independent variable when the independent variable is continuous. When the independent variable is categorical, *β̂* indicates the rate of change in stress from 1 category relative to the reference category. Adjusted *β̂* assumes that all other variables in the model are held constant.

^b^ Percentage of variability in the primary outcome explained by the factor for the univariate model. *R*^2 ^in the primary outcome explained by the set of factors for the multivariable model was 58.1.

^c^ The values of atmosphere range from 0 to 100, with 0 indicating calm and 100 indicating hectic or chaotic.

^d^ The values of the consequences construct range from 0 to 600, with 0 indicating not at all and 600 indicating to a great extent. It is composed from the total of the responses of 6 variables (pain, headache or eye strain, posture problems, sleep difficulties, anxiety or depression, and interference with work-life balance).

**Table 4.** Univariate and Multivariable Models for Burnout

Factor	Univariate Models				Multivariable Model	
	Unadjusted OR (95% CI)	*P* Value	AUC^a^	*R*^2^, %^b^	Adjusted OR (95 %CI)	*P* Value
EHR challenges construct variable, per 10-unit increase^c^	1.35 (1.15-1.59)	<.001	0.65	6.8	0.91 (0.71-1.17)	.48
Race/ethnicity	NA	>.99	0.51	0.02	NA	.19
White Non-Hispanic	1 [Reference]	NA	NA	NA	1 [Reference]	NA
Asian	0.99 (0.51-1.93)	.93	NA	NA	0.47 (0.17-1.30)	.83
Hispanic, any race	0.91 (0.42-1.99)	.87	NA	NA	0.37 (0.12-1.15)	.45
Other or unknown^d^	0.96 (0.36-2.53)	.98	NA	NA	0.39 (0.09-1.63)	.63
Office atmosphere, per 10-unit increase^e^	1.73 (1.42-2.11)	<.001	0.72	20.0	1.39 (1.10-1.75)	.006
Consequences construct variable, per 100-unit increase^f^	1.94 (1.56-2.40)	<.001	0.73	21.2	2.01 (1.48-2.74)	<.001
Primary care practice type	NA	.15	0.54	1.0	NA	.19
No	1 [Reference]	NA	NA	NA	1 [Reference]	NA
Yes	0.68 (0.41-1.14)	.15	NA	NA	0.58 (0.26-1.31)	.19
Procedural specialist practice type	NA	.04	0.54	2.1	NA	.34
No	1 [Reference]	NA	NA	NA	1 [Reference]	NA
Yes	2.21 (1.04-4.72)	.04	NA	NA	1.72 (0.57-5.21)	.34
Complex patient construct variable, per 50-unit increase^g^	1.20 (0.94-1.53)	.14	0.55	1.1	0.96 (0.68-1.36)	.83
Importance construct variable, per 1-unit increase^h^	0.83 (0.76-0.91)	<.001	0.65	7.5	0.91 (0.80-1.03)	.14
No. of years since completing training, per 1-unit increase	0.98 (0.96-1.00)	.02	0.58	2.8	0.98 (0.95-1.01)	.20

Abbreviations: AUC, area under the receiving operating characteristic curve; EHR, electronic health record; NA, not applicable; OR, odds ratio.

^a^ Accuracy rate of the model as it determines the discriminatory power of its estimation. For the multivariable model, the AUC was 0.81.

^b^ Percentage of variability in the primary outcome explained by the factor for the univariate models. *R*^2^ in the primary outcome explained by the set of factors for the multivariable model was 36.2%.

^c^ Created by averaging the response values from 9 questions yielding a possible range from 0 to 100 for the construct score.

^d^ Other category included Native American or Alaska Native, Native Hawaiian or Pacific Islander, Black or African American, or other.

^e^ Ranges from 0 to 100, with 0 indicating calm and 100 indicating hectic or chaotic.

^f^ Ranges from 0 to 600, with 0 indicating not at all and 600 indicating to a great extent. It is composed from the total of the responses of 6 variables (pain, headache and eye strain, posture problems, sleep difficulties, anxiety or depression, and interference with work-life balance).

^g^ Ranges from 0 to 300, with 0 indicating having a low percentage of complex patients and 300 indicating having very high percentage of complex patients. It is composed of the total of responses to 3 variables (patients with ≥3 complex medical problems, patients with complex or numerous psychosocial problems, and patients non-English speaking).

^h^ Ranges from 0 to 20, with 0 indicating a practice setting emphasizing slight or no importance and 20 indicating emphasizing great importance. It is composed from the total of responses to 5 variables (care for underserved populations, teamwork, information technology training, balancing professional and personal life, and productivity).

### Coping Strategies

Coping strategies for reducing stress felt to be associated with the EHR included talking with others (194 [68.8%]), exercise (192 [68.1%]), setting work boundaries (161 [57.1%]), discussing EHR messages with others rather than pinging electronic messages back and forth (149 [52.8%]), and writing shorter notes (142 [50.4%]). As a combined variable, coping strategies accounted for only 2.4% and 1.7% of the variability in stress and burnout respectively (data not shown). Setting boundaries (*β̂* = −0.02; *P* < .01) and taking breaks (*β̂* = −0.02, *P* = .006) were independently associated with reductions in overall stress, while exercise (aOR, 0.99; 95% CI, 0.98-1.00; *P* = .04) and taking breaks (aOR, 0.99; 95% CI, 0.98-1.00; *P* = .003) were associated with reductions in the odds of burnout.

### Factor Analysis of EHR Stress Items

We performed a statistical factor analysis using the varimax rotation method on the 9 EHR design and use factors listed in Table 2. We found that the first 2 statistical factors from the factor analysis accounted for 52.2% of the variability in EHR design and use items. We characterize these 2 factors as follows: (1) interference with patient care (eg, note bloat, interference with patient-clinician relationships, and notes geared toward billing) and (2) inefficient systems (eg, slow system response times, inability to navigate the system quickly, and excessive data entry). Thus, more than half of the variance in EHR issues associated with clinician stress and burnout stemmed from interference with patient care and inefficient EHR systems.

## Discussion

In this cross-sectional survey of 282 clinicians from 3 health systems, we identified 7 EHR design and use factors associated with high stress and burnout. These were information overload, slow system response times, excessive data entry, inability to navigate the system quickly, note bloat, interference with the patient-clinician relationship, fear of missing something, and notes geared toward billing. While previous studies have identified several of these EHR design and use items as challenging to clinicians,^9,24,25^ we believe this study is the first to show an association between these factors and objectively validated stress and burnout scales.

In this study, 45.0% of participants described symptoms of burnout, consistent with the findings of the national survey by Shanafelt et al^2^ in which 44% of physicians reported at least 1 symptom of burnout. The amounts of variation in stress and burnout associated with the EHR design and use factors listed in Table 2 were 12.5% and 6.8%, respectively. Thus, other sources of burnout aside from the EHR (such as lack of control of workload, chaotic environments, lack of attention to work-life balance, and ineffective teamwork) will also need to be addressed as medical practices seek to reduce burnout.

Many of the identified EHR design and use factors may be remediable through a combination of improvements by EHR vendors, local improvements by information technology personnel, and training of clinicians in the clinical environment. However, some of the identified factors may require higher-level actions on the part of clinic or governmental policy makers, for example, by allowing notes to be more geared toward clinical care than billing practices. Documentation requirements for billing purposes is an EHR design characteristic associated with both stress and burnout. The length of clinical notes has essentially doubled since the enactment of the Health Information Technology for Economic and Clinical Health Act.^26^ Physicians outside the United States are more likely to report satisfaction with their EHRs, where clinical documentation is significantly shorter and contains much less information in support of billing and compliance.^26^ The American Medical Informatics Association has recently called for a long-term strategy from the US Department of Health and Human Services to decouple clinical documentation from billing, regulatory, and administrative compliance requirements.^27^

Information overload may be associated with EHR design in which too much clinically unnecessary information is displayed. The aviation industry has a user interface design philosophy called *quiet dark*, where information is not displayed until something goes wrong or needs the pilot’s attention.^28^ In other words, the default state of all indicator lights is off during normal conditions. Applying this philosophy to EHR design could potentially reduce the amount of unnecessary data displayed based on particular users’ need and context, reducing the information overload problem. Arguably, the current state of EHR design is loud bright, where virtually all information, normal or otherwise, appears in relatively the same manner regardless of its importance to the clinician or patient. Although abnormal results from laboratory tests are highlighted, all normal values are typically displayed and occupy the same amount of space and are given the same prominence as abnormal results. Given the proliferation of standardized templates as a time-saving tool for data entry, the amount of unnecessary, repetitive, normal information (ie, note bloat) is increasing vs a design where an economy of information relevant to the patient’s current needs and context is used.^29^

The data entry problem has created the scribe movement and produced promising results, at least in terms of clinician and patient satisfaction.^30^ However, scribes only help with data entry during office visits and not with EHR tasks at other times and in other venues. A more comprehensive approach is to use specially trained medical assistants (MAs) to relieve the clinician from clinic tasks (eg, responding to routine in-basket messages, refilling some prescriptions per protocol, completing paperwork). Before the clinician meets the patient, the MA completes prework (eg, medication reconciliation, review of systems, documentation of chief concern, and any protocolized clinical measurements, such as peak flows or pulse oximetry). The MA scribes during the clinician encounter, and after the clinician leaves the room, the MA can review the plan of care, deliver patient education, process referral requests, and schedule follow-up appointments.^31^

Some of the troublesome EHR design and use factors, such as the inability to navigate the system quickly, are attributable to computer-human interaction problems. In fact, most of the current EHR user interface designs are still based on 2-dimensional paper metaphors (eg, tabs, flowsheets, tables, and forms) and do not take advantage of the potential of graphics capabilities now in the most basic computers.^32^ More research to determine what display metaphors beyond paper are most efficient could help. Complaints of interference with the patient-clinician relationship is evidence that clinicians are troubled by their excessive focus on the screen rather than the patient. While most studies have shown the presence of the EHR in the exam room does not adversely affect patient satisfaction,^9,33,34^ clinicians feel that EHRs requiring clinically irrelevant data entry take away from their relationships with their patients.^35^ Our study shows that this is significantly associated with clinician stress and burnout.

The proportion of clinicians reporting pain (47.5%) and posture issues (51.1%) attributed to EHR use was high. Ergonomics are rarely addressed in most clinical settings. Clinicians often must work at several workstations, with different heights and seat structures. Collaboration with employee health groups skilled at ergonomics could potentially have a substantive effect on the health outcomes of our clinician workforce.^36^ This is an area ripe for further quality improvement studies.

Coping strategies clinicians suggested to reduce EHR-associated stress included exercise (used by 68.1% of our sample), verbally discussing issues with other clinicians (68.8%), and setting boundaries for work while at home (57.1%). Setting boundaries, exercise, and taking breaks were significantly associated with reductions in overall stress and burnout and may be useful components to incorporate into stress reduction interventions. It is not clear how many of these strategies clinicians actually used or how effective they were at using them.

### Strengths and Limitations

The strengths of this study include surveying a diverse group of clinicians, including academic, community-based, and rural institutions and practices, physicians and advanced practice clinicians, and a mix of specialists and nonspecialty ambulatory care clinicians. In addition, the list of the EHR design and use factors the clinicians rated in the survey was defined by clinicians in multi-institutional focus groups.^9^ The survey response rate (44.1%) was reasonable for large clinician-based studies with no financial incentive. The design of the instrument included questions previously validated in studies of physicians about stress and burnout.

This study has limitations, including its cross-sectional nature and the use of self-reported metrics. One needs to consider response bias, given the 44.1% response rate. The relatively modest sample size limits validity. As respondents came from only 3 institutions, these results may not be more widely generalizable. The mapping of the paper instrument’s Likert scales to the REDCap slider bars scale may have introduced some bias. Despite using validated instruments to measure burnout and stress, the survey relied on the respondents’ own definitions. Self-reported metrics may underrepresent the numbers at risk. As Knox et al^37^ found, a self-defined, single-item burnout measure identified significantly fewer physicians most at risk of burning out compared with the Maslach Burnout Inventory. All respondents were grouped together for this analysis, which does not account for possible intragroup differences, such as between physicians and advanced practice clinicians.^37^

## Conclusions

Stress and burnout associated with EHRs is prevalent and may be at least partly remediable at the local level. The issues identified in our list of EHR-associated challenges may provide designers, government regulators, and clinical leaders with targets for improvement of EHR design. Other work conditions are associated with stress and burnout in clinicians and deserve equal attention.
